# Oxysterol sulfates in fluids, cells and tissues: how much do we know about their clinical significance, biological relevance and biophysical implications?

**DOI:** 10.1042/EBC20230090

**Published:** 2024-12-04

**Authors:** Ana Reis, Irundika H.K. Dias

**Affiliations:** 1 REQUIMTE/LAQV, Departamento de Química e Bioquímica, Faculdade de Ciências, Universidade do Porto, Rua do Campo Alegre, 4169-007 Porto, Portugal; 2 Aston Medical School, Aston University, Birmingham, B4 7ET, U.K.

**Keywords:** biofluids, cholesterol sulfate, membrane fluidity, neurodegenerative diseases, oxysterols

## Abstract

Oxysterol sulfates are emerging as key players in lipid homeostasis, inflammation and immunity. Despite this, knowledge on their basal levels in fluids, cells and tissues and any changes associated with age, gender and diet in health and disease; as well as their spatio-temporal distribution in cell membranes and organelles have been greatly hampered by the lack of commercially available pure synthetic standards. Expansion of the panel of pure oxysterol sulfates standards is pivotal to improve our understanding on the impact of oxysterol sulfates at the membrane level and their role in cellular events. While the clinical significance, biophysical implications and biological relevance of oxysterol sulfates in fluids, cells and tissues remains largely unknown, knowledge already gathered on the precursors of oxysterol sulfates (e.g. oxysterols and cholesterol sulfate) can be used to guide researchers on the most relevant aspects to search for when screening for oxysterol sulfates bioavailability in (patho)physiological conditions which are crucial in the design of biophysical and of cell-based assays. Herein, we provide a review on the brief knowledge involving oxysterol sulfate and an overview on the biophysical implications and biological relevance of oxysterols and cholesterol sulfate useful to redirect further investigations on the role of oxysterol sulfates in health and disease.

## Introductory perspective

Oxysterol sulfates are members of the diverse family of lipid sulfates. a heterogeneous class of lipids distributed across a wide mass range with distinct structural motifs containing sulfate group in its structure [1].

**Figure 1:**
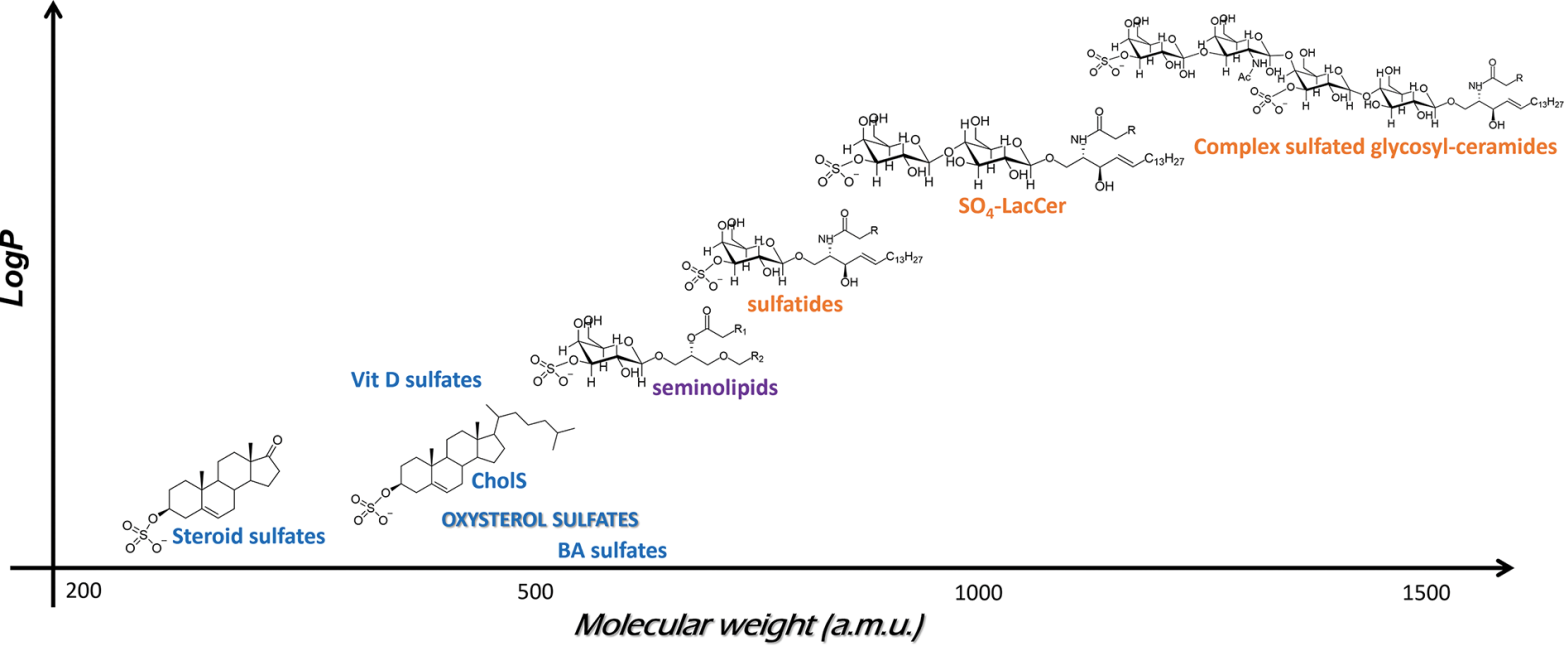
Structures of sulfate-containing lipids. Structure of sulfate-containing lipids identified in human biological samples bearing a steroid-backbone (blue), a glycerol-backbone (purple) and a sphingosine-backbone (orange). LogP predicted values (ALOGPS model) were taken from Human Metabolome DataBase (HMDB). Substitution of ceramide with increasing number of sugar residues confers lipid sulfates increasing solubility and distribution across a wide mass range.

In mammals, lipid sulfates (Figure 1) can be sub-divided in those bearing a steroid-backbone (e.g. cholesterol sulfate [CS], steroid hormones, and bile acids), glycerol-backbone (e.g. seminolipids) and sphingosine-backbone (e.g. sulfatides and higher glycosylated-ceramides). Interestingly, the insertion of glycosyl residues in lipid sulfates occurs by two distinct intermediates, via uridine diphosphate (UDP)-glucose with formation of glucosyl-ceramides (GlcCer), the precursor of major glycosphingolipids, and via UDP-galactose leading up to the formation of seminolipids and sulfatides [2]. Lipid sulfates are ubiquitous to the whole body and distributed across fluids, cells and tissues [1] and during their analysis are often accompanied by other lipid-like sulfated metabolites [1]. Due to their relevance in human reproduction, growth development and sports performance, steroid sulfates (e.g. sex hormones, vitamin D and bile acids) are the most widely studied among lipid sulfates and their basal levels and changes with age, gender and disease are fairly well-characterised [3,4]. On the other hand, oxysterol sulfates, remain largely understudied.

**Figure 2:**
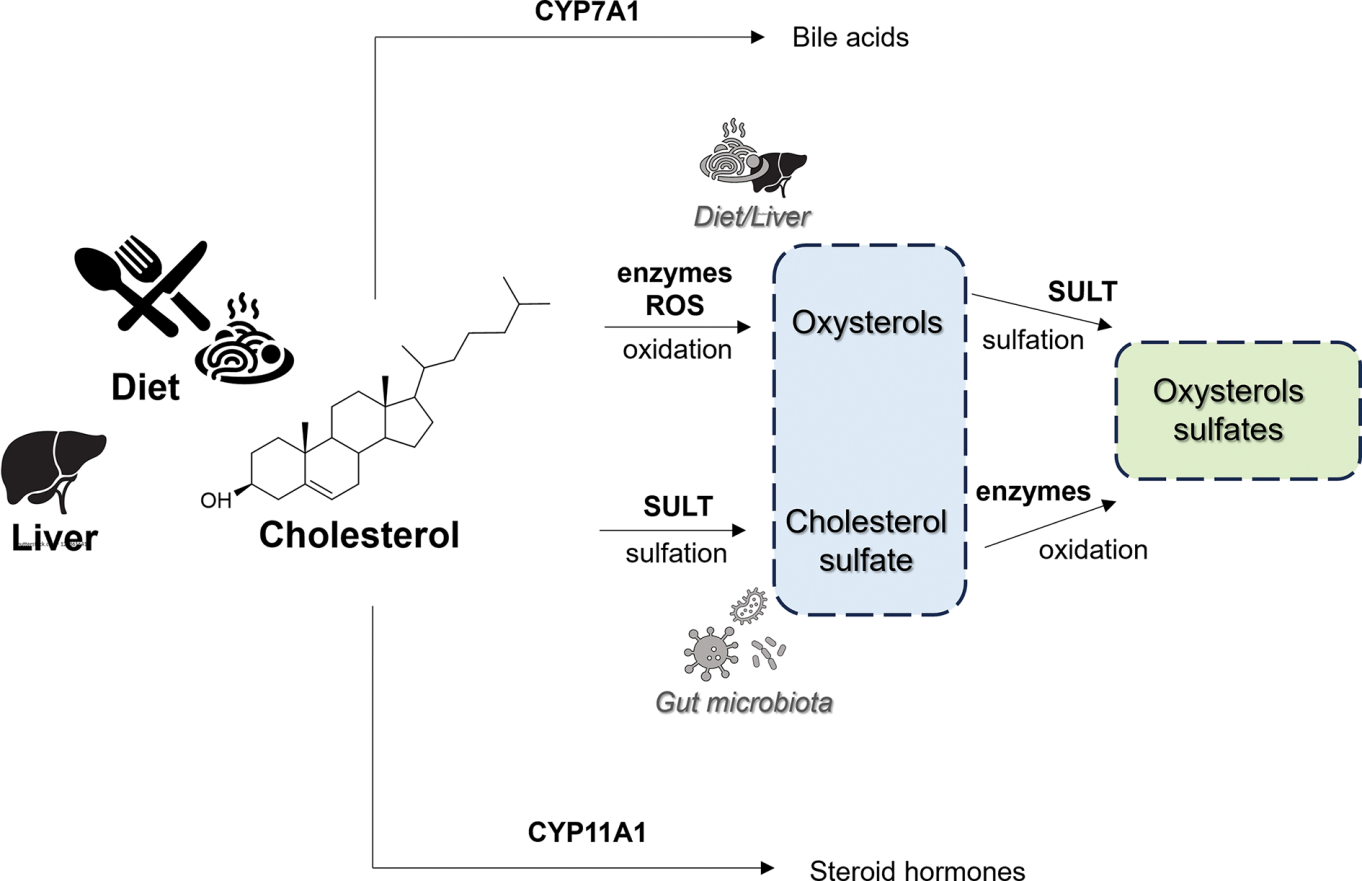
*In vivo* formation of oxysterol sulfates (green box) from its precursors (blue box). Oxysterol sulfates can be formed via sulfation of oxysterols formed by oxidation of cholesterol, or via oxidation of cholesterol sulfate formed by sulfation of cholesterol.

Oxysterol sulfates can arise *in vivo* by two main routes (Figure 2). Oxysterol sulfates formed by (auto)oxidation of dietary and liver cholesterol via enzymatic or non-enzymatic routes with formation of oxysterols where they can undergo sulfonation by cytosolic enzyme SULT2B1b [5,6] or alternatively, being CS a better substrate for CYP27A1 than cholesterol [7], they can be formed enzymatically by (auto)oxidation from endogenous CS or host microbiota CS [8].

**Figure 3:**
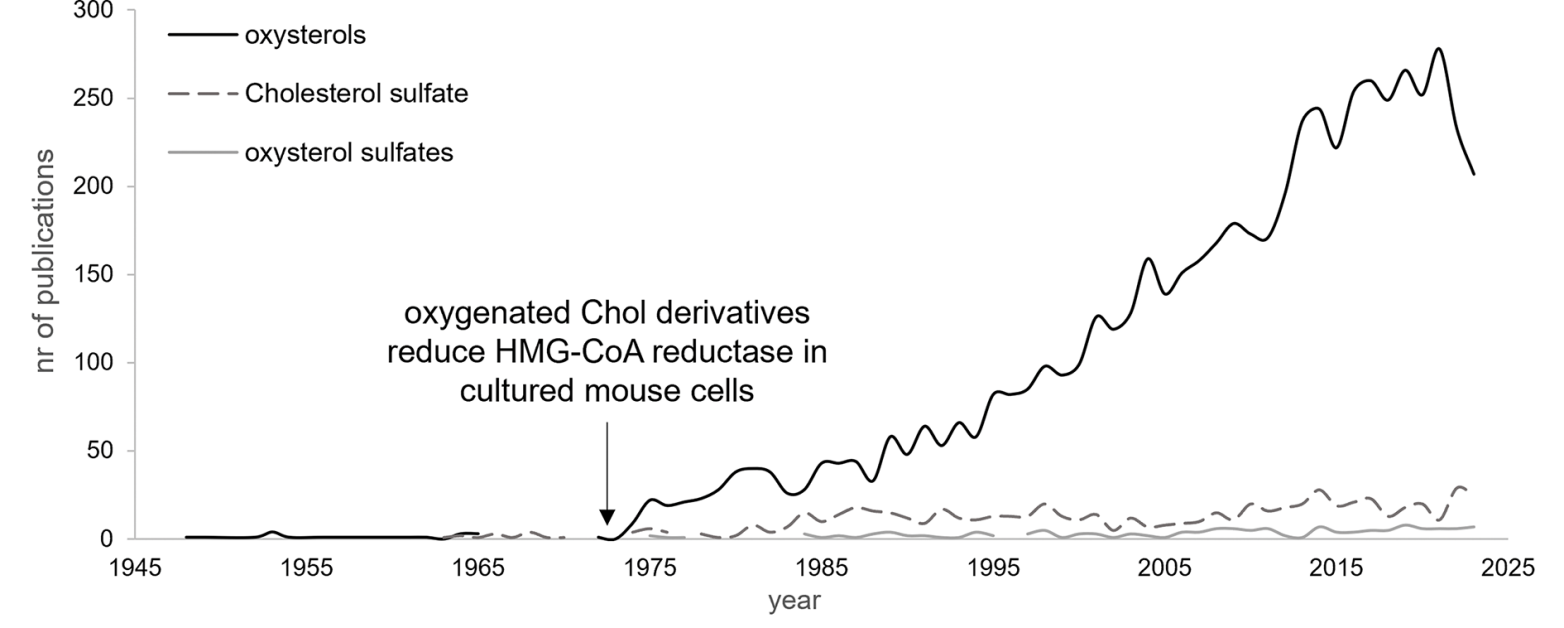
Number of publications on oxysterol sulfates. Number of publications retrieved using the search string ‘oxysterol sulfates’ and its precursors ‘oxysterols’ and ‘cholesterol sulfate’ encompassing the period from 1945 to 2023 (PubMed search, assessed 3 January 2024).

In view of the reported raised levels of oxysterols [9–11] and cholesterol sulfate [12,13] in disease, they hint to the likely *in vivo* accumulation of oxysterol sulfates rendering these compounds valuable prognostic potential as markers in risk prediction and disease stratification. However, although the initial findings of sulfate conjugates of C27 steroids (oxysterol sulfates) date back to the 1970s reported in the plasma and urine of cholestatic patients [14], investigations have mainly focused on oxygenated sterols (Figure 3) prompted by the discovery that oxygenated sterols, but not exogenous cholesterol or other metabolically related steroids, depressed the activity of 3-hydroxy-3-methylglutaryl-coenzyme A (HMG-CoA) reductase [15].

Since then, and contrary to the widely studied oxysterols, its sulfated derivatives have received very little attention over the years and their prognostic value remains, to date, largely unexplored. To date, very little is known about: (1) the oxysterol sulfate ‘signature’ and basal levels in fluids, cells and tissues, crucial considering the heterogenous panel of oxysterols reported in serum, urine and cerebrospinal fluids (CSFs) [11,16]; (2) the carriers of oxysterol sulfates in circulation, relevant considering the significantly raised levels of CS in low-density lipoproteins (LDLs) of recessive X-linked ichthyosis (RXLI) patients but not in other lipoprotein populations [17]; (3) the changes associated with age, gender, diet and disease, considering the reported age- and gender- dependence in CS and oxysterols levels [11,18]; and finally (4) the spatio-temporal distribution across cell membrane and organelles, crucial considering the wide distribution of oxysterols across cell organelles [19]. Bearing this in mind, it is rather puzzling why the analysis of oxysterol sulfates in biological samples has not yet been routinely implemented in research laboratories considering the technological advances made in the development of user-friendly mass spectrometers equipped with fast scan rates, high sensitivity and high resolution, hugely responsible for the popularity of Omic platforms in clinical research, and the automation of lipid analysis pipeline (e.g., lipid extraction, LC-MS data acquisition, data pre-processing, lipid identification and quantification) which are already in place in most large-scale lipidomic laboratories.

One of the reasons for the lack of interest in the study of oxysterol sulfates could be related to the reported very low levels at which oxysterol sulfates [16,20], as well as their precursors (e.g., oxysterols and cholesterol sulfate), occur. When compared with cholesterol the *ex vivo* levels reported for oxysterol-to-Chol ratio are of 1:1000 and CS-to-Chol ratio of 1:500 [21,22] suggesting that these molecules may have little physiological relevance. Their low physiological levels are determined by the rate of *in vivo* formation and turnover to other molecules (e.g., bile acids, steroid hormones, cholesterol, and other sterols in the case of oxysterols) and non-selective reversal by steroid sulfatase (in the case of cholesterol sulfate and oxysterol sulfates).

Although very little is known about the distribution of oxysterol sulfates in cell membranes and other organelles, their concentration values, or their impact on the biophysical properties of cell membranes are scarce. However, their precursors (e.g., oxysterols and cholesterol sulfate) are intrinsic components of cell membrane and other organelles [19,21]. Because of the oxidative environment in mitochondria and the intracellular occurrence of CYP and cytosolic SULT2 enzymes [5,23], it is plausible to infer the occurrence of oxysterol sulfates as constituents of cell membranes and other organelles. In addition, recent biophysical studies have shown that as integral components of cell membranes, oxysterols and cholesterol sulfate affect the structure, organisation and dynamics of lipid bilayers changing the physical properties of cell membranes [24–27]. Changes to the dynamics and organisation of membranes, modify the oxysterol–phospholipid–protein interactions and the cross-talk of oxysterol-sensing proteins highlighting the biological importance of these compounds in membrane-mediated cell events in (neuro)inflammatory responses, immune adaptation signalling and cell function.

Herein, we overview the current knowledge on oxysterol sulfates and provide a critical evaluation on the caveats and challenges surrounding investigations on oxysterols and cholesterol sulfate which could be used to improve our understanding on the *in vivo* clinical significance, biological relevance and biophysical implications of oxysterol sulfates.

## *In vivo* oxysterol sulfates: clinical relevance and biological significance

While in theory, the structural diversity of oxysterol sulfates may be similar to that reported for oxysterols, in practice only a handful of oxysterols sulfates has been identified *ex vivo* in biofluids, namely the 24-, 25- and 26-hydroxy-cholesterol sulfates using LC-MS approaches [20,28] together the 26-hydroxy-cholesterol-27-sulfate (26OHC27S) [20], as well as the 25-hydroxy-cholesterol-3-sulfate (25OHC3S) and the 25-hydroxy-cholesterol-3,25-disulfate (25OHCDS) in *in vitro* cultured rat hepatocytes [29,30]. Interestingly, assuming that the ionisation efficiency of oxysterol sulfates isomers under gas-phase fragmentation is similar, then the distinct LC-MS profile observed in serum extract of RLXI patient and a cholestatic baby and the dissimilar relative abundance [20] suggests that the panel of predominant oxysterol sulfates may be an individual trait.

In fact, work on circulating and excreted oxysterols revealed that 26-HC predominates in circulation in healthy adults [31] whereas the 24-HC isomer predominated over 26-HC isomer in CSF [16] and the 22-HC was the main urinary oxysterol found in neonates [11]. With very few exceptions [31–33], most of the screening studies focus on a small panel of oxysterols in a small number of donors [9,10,34–36] preventing from getting the full image of oxysterol panel in biological samples. In a recent study conducted on an extended panel of enzymatically and non-enzymatically generated oxysterols in plasma and serum of a large cohort (*n*=2282) of women with breast cancer diagnosis, the authors found higher levels of six oxysterols with predominance of 7-hydroxy-cholesterol (7-HC), 7-keto-cholesterol (7-KC) and 26-hydroxy-cholesterol (26-HC) in circulation [33]. While the increased levels of these oxysterols in women with breast cancer may be biased due to the lack of standardisation in sterol analysis pipeline (e.g., sample collection, analyte isolation, extract storage and quantification procedure [9,37]), the results suggest a disease-related dependence on the predominant oxysterol in circulation highlighting the importance of comprehensive screening studies in sterol research with the inclusion of both enzymatically and non-enzymatically-generated derivatives. In view of this, the use of sentences such as ‘*one of the most important oxysterol molecules derived from cholesterol…*’ or ‘*the most abundant oxysterols in the human body…*’ are ambiguous and misleading to the non-expert audience and should be avoided in published manuscripts.

Currently, quantification of oxysterol sulfates can be achieved against a calibration curve using available analytical standards [16] however, the only analytical synthetic standard commercially available for targeted LC-MS quantification purposes is the 25OHC3S (https://www.sigmaaldrich.com/PT/en/product/avanti/700017p). Although, no differences are foreseen in the ionisation efficiency between oxysterol sulfate isomers bearing hydroxy and sulfate groups in different positions of the sterol rings, in reality the small differences reported in the peak area counts (cps) of several hydroxy-cholesterol derivatives [9] suggests this may not be the case. These findings highlight the need to engage in collaborative efforts with organic chemists to expand the panel of pure synthetic standards.

To the best of our knowledge, only the 27-hydroxy-cholesterol-3 sulfate (27OHC3S, otherwise known as (25R)-26-hydroxycholesterol-3-sulfate) [37]) was quantified by targeted LC-MS strategies in serum samples from patients with steroid sulfatase deficiency [20]. More recently, Dias and colleagues (2022) reported on the total levels of oxysterol sulfates in AD patients by direct infusion in Qtrap MS instrument and found that the levels of oxysterol sulfates in both brain tissue and CSF sample were lower in AD patients when compared with controls [16]. Even though the values reported in AD patients were significantly lower (ng/L) than those reported previously in serum samples (ng/ml) [20] work on oxysterols has already shown that, given the dynamic efflux of brain oxysterols into circulation [38], less than 1% of the daily production of the brain oxysterols enters the CSF and approximately 99% enters the circulation via the blood–brain barrier (BBB) [39]. For this reason, and given that plasma oxysterol levels can be altered by exogenous (diet) or endogenous (enzyme and microbiota) cholesterol metabolism, it is believed that CSF levels more closely reflect the integrity of BBB and more consistently relate to neuronal diseases [40].

Although the panel and basal levels of circulating oxysterols sulfates and the spatio-temporal distribution in cells, organelles and tissues, and any alterations introduced by age, gender and diet in health and disease remain elusive, the values of oxysterol sulfates described in biological samples report to nanomolar range [16,20]. In spite of this, *in vitro* cell-based research has shown that oxysterol sulfates may act as epigenetic regulators, agonists, and antagonists of DNA methyl-transferases, evidencing their function of global regulation through epigenetic modification [41]. However, most of these studies cell-based assays were incubated with supraphysiological concentrations (3–25 μM) of 25HC3S [29,42,43], values that far exceed the reported ones in the literature and thus with poor biological relevance. Future studies aimed to accurately corroborate oxysterol sulfate levels in biofluids, cells and tissues are advisable, providing the basis for more relevant and realistic conditions when studying the (patho)physiological role of oxysterol sulfates in (neuro)inflammation.

## *In vitro* oxysterol sulfates: biophysical implications to biomembranes

Of more than 80 sterols described in nature, only a few, including cholesterol, ergosterol, and some phytosterols, are crucial components of cellular membranes. Most of the remaining sterols, including the sub-class of steroid sulfates, are metabolites or signalling molecules (hormones and bile acids) [44].

Cholesterol is a prominent sterol of mammalian cell membranes. Embedded in lipid bilayers, cholesterol is typically associated with sphingo- and glycolipids forming nanodomains known as lipid rafts that serve as anchoring points for transmembrane proteins and crucial for proper signalling and trafficking mediated events [45]. At the biophysical level, cholesterol has a ‘condensing’ and rigidifying effect on biomembranes increasing the membrane order leading to increased bilayer thickness and decreased membrane permeability to water and solutes [46,47]. Unlike cholesterol, its sulfated conjugate does not diffuse freely across cell membranes. The addition of a hydroxyl and sulfate groups confers an amphiphilic character to the hydrophobic cholesterol modifying the interface properties of membranes and consequently its biophysical properties. Due to its charged and fully hydrated sulfate headgroup, the sufate group is large enough to act as a spacer between phospholipid molecules rendering cholesterol sulfate (CS) a more surface-localised position within biomembranes [48]. Studies with artificial biomembranes have shown that the presence of CS induces a slight ordering effect (to a less extent than Chol) [48] able to induce a greater decrease in the transition temperature of PC membrane models when compared with Chol [49].

Similar to other sulfate-based lipids, such as sulfoglycoceramides (SGC) and sulfoglycoglycerolipids (SGG), CS is located at the water-lipid interface of membranes contributing to the overall membrane net surface charge [48]. CS and other sulfate-based lipids (SGC and SGG) gather at sphingolipid-cholesterol-rich domains (lipid rafts) contributing to membrane stabilisation [50] and implicated in mediating membrane-driven cell-cell processes such as sperm-egg interaction [13], skin differentiation [51], membrane fusion [52] and platelet-cell adhesion [53].

**Figure 4:**
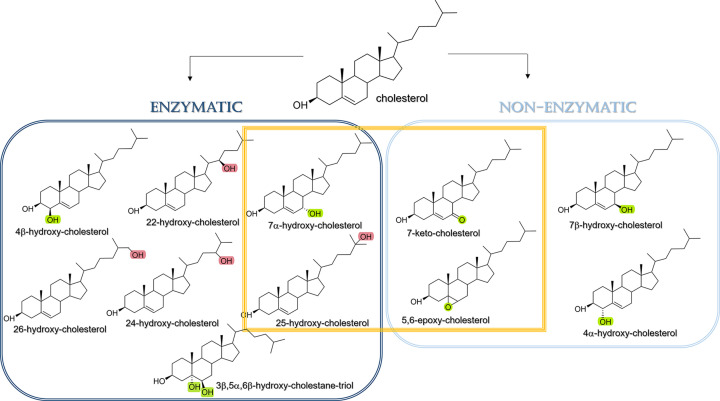
Structures of main oxysterols found *in vivo*. Structures of main oxysterols formed via enzymatic pathways (dark blue) and non-enzymatic pathways (light blue). Oxysterols formed by both routes are delimited by orange oval circle. Oxidative modifications at the rings (ring-oxidised, green) and at the iso-octyl hydrocarbon tail (tail-oxidised, pink) are highlighted.

On the other hand, oxysterols diffuse across lipid bilayers including polarised epithelial membranes (e.g., BBB) [38]. Insertion or replacement of membrane Chol by oxysterols decreases the molar fraction of Chol to the membrane’s composition changing the structure and dynamics of lipid bilayers [24–26,54]. As cholesterol is oxidised with formation of oxysterols and membrane cholesterol is replaced, how do cells sense and adapt to these changes? One of the accepted mechanisms proposes that these changes are accompanied by changes to the biophysical properties of membranes triggering downstream biochemical events [55]. With this in mind, how do oxysterols affect cell membranes? Work aimed to assess the impact of oxysterols on artificial membranes found that the position of the hydroxy group was particularly relevant [24,25]. Combining spectroscopic measurements and atomistic molecular dynamic simulations on a panel of enzymatically- and radical-derived oxysterols, Kulig and colleagues found that oxysterols with the hydroxyl group within the ring- behaved differently than those with hydroxyl group at the hydrocarbon tail (Figure 4).

**Figure 5:**
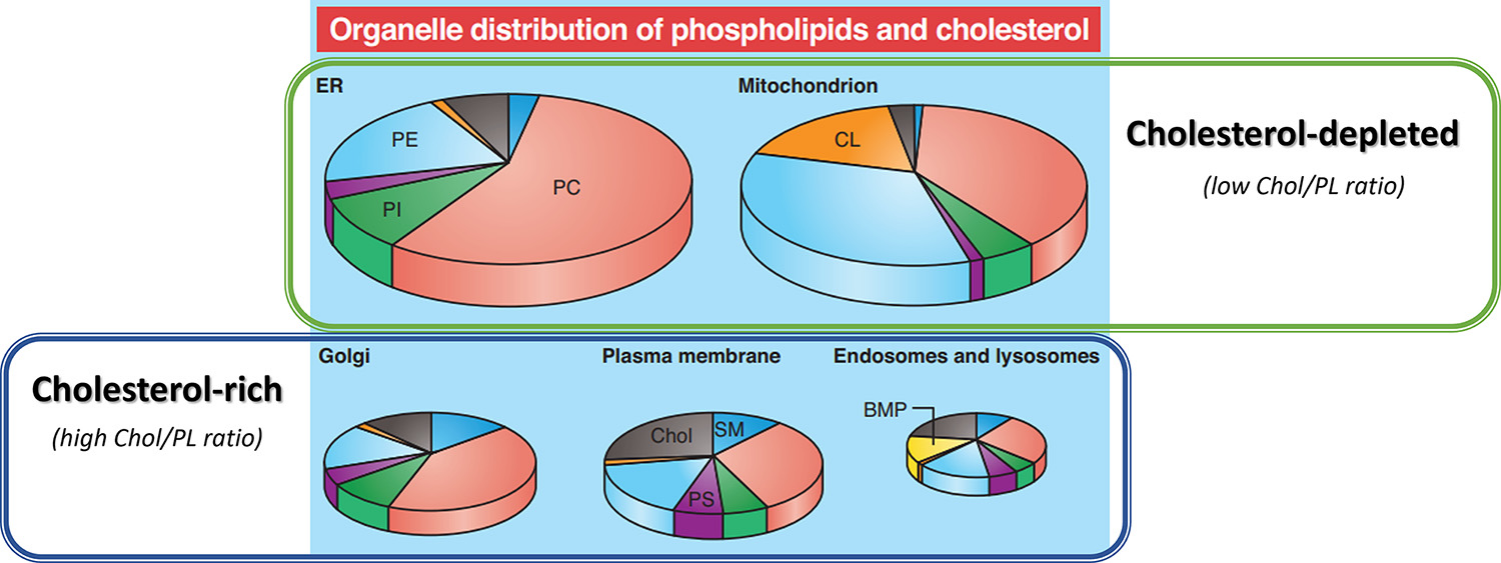
Sub-cellular distribution of main phospholipid classes and cholesterol in plasma membrane and other cell organelles. (Red) PC: Phosphatidylcholine; (light blue) PE: Phosphatidylethanolamine; (purple) PS: Phosphatidylserine; (green) PI: Phosphatidylinositol; (dark blue) SM: sphingomyelin; (yellow) BMP: bis(monoacylglycero)phosphate; (orange) CL: cardiolipin; (grey) Chol: cholesterol (adapted from van Meer & de Kroon “Lipid map of the mammalian cell” published in J Cell Sci. (2011) 124, 5. Reprinted with permission from The Company of Biologists, Ltd).

While the presence of ring- and tail- oxysterols induce an increase in the order of POPC lipid bilayers (stiffening effect), ring-oxidised oxysterols (such as 7-HC) exhibit augmented effect in the ordering of acyl chains when compared with tail-oxidised oxysterols (such as 25-HC), leading to increased bilayer thickness and decreased surface area in a manner similar to Chol. In opposition, the smaller ordering effect induced by tail-oxidised oxysterols to the membrane means their presence in artificial PC membranes display a slight permeability [24,25,56]. Data from atomistic simulations suggest that tail-oxidised oxysterols, unlike ring-oxidised, accommodate inside the lipid bilayer in a fluctuating manner [57] where the nearly perpendicular tilted orientation with respect to bilayer enables the tail- and the 3β-hydroxyl groups to interact with the phospholipid polar headgroups at the water:lipid interface [24,58]. Further studies with tail-oxidised 25-HC isomer using various 25-HC and cholesterol molar ratios revealed that the behaviour of tail- oxysterols shows distinct behaviour depending on the content (molar %) of cholesterol [26]. The distinct behaviour of oxysterols in cholesterol-depleted and cholesterol-rich membranes [26] is of significant physiological relevance considering the content of cholesterol in mammalian membranes ranges from 5–10% in mitochondria and endoplasmic reticulum (Figure 5) to 30–50% in plasma membranes, and over 50% in the brain myelin sheath [59,60].

While not yet commercially available, the synthesis of tail- and ring-oxidised oxysterol sulfate standards is pivotal to understand the role of these compounds at the membrane level. The behaviour of ring- and tail- oxysterols in Chol-poor and Chol-rich model membranes herein overviewed provides a starting point to take into account in future investigations on the biophysical implications of oxysterol sulfates in cholesterol-rich plasma membranes (e.g., endothelial and myelin sheath) or in cholesterol-depleted membranes (e.g., mitochondrion and endoplasmic reticulum). Likewise, understanding how changing levels of oxysterol sulfates likely to occur with age and disease have on the conformation and behaviour of oxysterol-binding proteins (OBPs) operating at the membrane contact sites, their performance on intracellular lipid homeostasis and trafficking and on other membrane-driven signalling processes [61] is of the utmost importance.

## Concluding remarks

To date, the clinical significance, biological relevance and biophysical implications of oxysterol sulfates in cell membranes and other organelles remains poorly understood by the scientific community. To improve our understanding on these aspects particularly in the context of (neuro)inflammatory diseases requires the concerted and integrated effort of professionals from different fields of research including organic chemists pivotal in the development of Reference Standard Material; clinicians and health technicians together with analytical chemists for the implementation of Reference Standard Protocols tailored to the screening and quantification of circulating and excreted oxysterol sulfates in biofluids, cells and tissues so that membrane biophysics and cell biologists may decipher the role of oxysterol sulfates in membrane organisation, and cell metabolism and trafficking.

## Summary

- Raised levels of circulating oxysterols and cholesterol sulfate in disease point to the *in vivo* accumulation of oxysterol sulfates.
- High levels of oxysterol sulfates were reported in CSF and brain tissue in AD patients.
- The diagnostic value of oxysterol sulfates in risk prediction and disease stratification relies on improved knowledge of basal values in health and disease.
- Pure oxysterol sulfates synthetic standards are not yet commercially available.
- Expansion of standards panel is key to improve our understanding on the relevance of these compounds in (neuro)inflammation.

## Open Access

Open access for this article was enabled by the participation of Aston University in an all-inclusive *Read & Publish* agreement with Portland Press and the Biochemical Society under a transformative agreement with JISC.

## Abbreviations

7-HC: 7-hydroxy-cholesterol
26-HC: 26-hydroxy-cholesterol
7-KC: 7-keto-cholesterol
BBB: blood–brain barrier
CS: cholesterol sulfate
CSF: cerebrospinal fluid
LDL: low-density lipoprotein
OBP: oxysterol-binding protein
RXLI: recessive X-linked ichthyosis
SGC: sulfoglycoceramides
SGG: sulfoglycoglycerolipids
